# Synergy between Readthrough and Nonsense Mediated Decay Inhibition in a Murine Model of Cystic Fibrosis Nonsense Mutations

**DOI:** 10.3390/ijms22010344

**Published:** 2020-12-31

**Authors:** Daniel R. McHugh, Calvin U. Cotton, Craig A. Hodges

**Affiliations:** 1 Department of Genetics and Genome Sciences, Case Western Reserve University, 10900 Euclid Ave, Cleveland, OH 44106, USA; dmchugh@convelotx.com; 2 Department of Pediatrics, Case Western Reserve University, 10900 Euclid Ave, Cleveland, OH 44106, USA; ccotton@cff.org; 3 Department of Physiology and Biophysics, Case Western Reserve University, 10900 Euclid Ave, Cleveland, OH 44106, USA

**Keywords:** cystic fibrosis, readthrough, nonsense-mediated decay, organoid, premature termination codon, aminoglycoside, intestinal organoid

## Abstract

Many heritable genetic disorders arise from nonsense mutations, which generate premature termination codons (PTCs) in transcribed mRNA. PTCs ablate protein synthesis by prematurely terminating the translation of mutant mRNA, as well as reducing mutant mRNA quantity through targeted degradation by nonsense-mediated decay (NMD) mechanisms. Therapeutic strategies for nonsense mutations include facilitating ribosomal readthrough of the PTC and/or inhibiting NMD to restore protein function. However, the efficacy of combining readthrough agents and NMD inhibitors has not been thoroughly explored. In this study, we examined combinations of known NMD inhibitors and readthrough agents using functional analysis of the *CFTR* protein in primary cells from a mouse model carrying a G542X nonsense mutation in *Cftr*. We observed synergy between an inhibitor of the NMD component SMG-1 (SMG1i) and the readthrough agents G418, gentamicin, and paromomycin, but did not observe synergy with readthrough caused by amikacin, tobramycin, PTC124, escin, or amlexanox. These results indicate that treatment with NMD inhibitors can increase the quantity of functional protein following readthrough, and that combining NMD inhibitors and readthrough agents represents a potential therapeutic option for treating nonsense mutations.

## 1. Introduction

Ten percent of heritable diseases are caused by nonsense mutations [1,2]. Nonsense mutations are single nucleotide alterations which generate premature UAG, UAA, or UGA termination codons (PTCs) in mRNA transcripts. During translation, PTCs are decoded by eukaryotic release factor 1 (eRF1) rather than an aminoacyl-tRNA. eRF1 is preferentially incorporated into the A site of the ribosome, causing hydrolysis of the ester bond linking the elongating polypeptide chain to the P-site tRNA [3]. This results in premature termination of translation and generates truncated proteins which are often nonfunctional or have deleterious dominant-negative properties [4,5]. Expending of energy and resources on producing dysfunctional protein is a wasteful procedure for the cell; therefore, nonsense-carrying transcripts are degraded prior to translation by nonsense-mediated decay (NMD) mechanisms. Failure to complete the first round of translation signals the recruitment of NMD machinery which degrades the mutant transcripts, preventing future attempts at translation [6,7,8].

Aminoglycoside antibiotics have been found to facilitate readthrough of PTCs by reducing the energy required to create a stable mRNA to tRNA bond at the ribosome [1,9,10,11,12]. This allows incorporation of a near-cognate aminoacyl tRNA, continuing translation. Aminoglycoside treatment is associated with nephrotoxicity and ototoxity in patients [13]; therefore several groups have identified alternative readthrough agents, such as PTC124 [14] and escin [15], through high throughput screening. However, these compounds have limited readthrough properties compared to more efficacious aminoglycosides [16,17,18]. The rarity of readthrough is due in part to degradation of mutant mRNA by NMD, which reduces the quantity of mRNA that is subject to readthrough. Increasing the quantity of mutant mRNA using inhibitors of NMD is an attractive therapeutic option to make readthrough more effective. To this end, several compounds which inhibit NMD have been identified, including amlexanox [19], NMDI-14 [20], and a small molecule inhibitor of the NMD component SMG-1 (SMG1i or inhibitor of suppressor with morphological effect on genitalia 1) [21]. However, a thorough examination of synergy between currently known readthrough agents and NMD inhibitors has not been performed.

Cystic fibrosis (CF) is a heritable disease which can be caused by nonsense mutations. CF arises from mutations in the cystic fibrosis transmembrane conductance regulator (*CFTR*) gene, which expresses an apically localized anion channel in epithelial tissues. Loss of *CFTR* function causes dehydration of epithelial surfaces, leading to accumulation of viscous mucus. Patients with CF suffer from a wide range of symptoms, including pulmonary dysfunction [22], intestinal maladies [23], and reduced growth [24]. CF is a fatal disorder, with the most common cause of mortality being lung failure [25]. Small molecule therapies which restore function to *CFTR* have been identified for several *CFTR* mutation types [26,27,28], but an effective therapy which restores function to *CFTR* containing nonsense mutations is unavailable.

Recently, we generated a mouse model of the G542X nonsense mutation in *Cftr* [29], the most common CF-causing nonsense mutation [9,30]. CFTR function can be examined in intestinal organoids harvested from G542X mice by measuring *CFTR*-dependent forskolin-induced swelling (FIS) [31,32]. We have previously demonstrated that G542X organoids are able to undergo FIS following treatment with G418. A number of additional aminoglycosides are available which have reduced readthrough potency and toxicity relative to G418 [9,16,33,34,35,36]. Here, we examine G418, several alternative aminoglycosides, and identified non-aminoglycoside readthrough agents in combination with known NMD inhibitors using functional analyses of CFTR in primary cells from G542X mice. We found that SMG1i treatment can increase the amount of functional CFTR produced in G542X intestinal organoids following readthrough by the aminoglycosides G418, gentamicin and paromomycin, but not with tobramycin, amikacin or the readthrough agents PTC124 or escin. In addition, inhibition of SMG-1 synergizes with aminoglycoside readthrough to improve CFTR function in primary airway epithelial cell cultures, suggesting that NMD inhibition and readthrough synergy can occur in both intestinal and airway cells.

## 2. Results

### 2.1. Intestinal Organoid FIS Is a More Biologically Relevant Detector of Readthrough than Cell-Based Reporters

**Figure 1:**
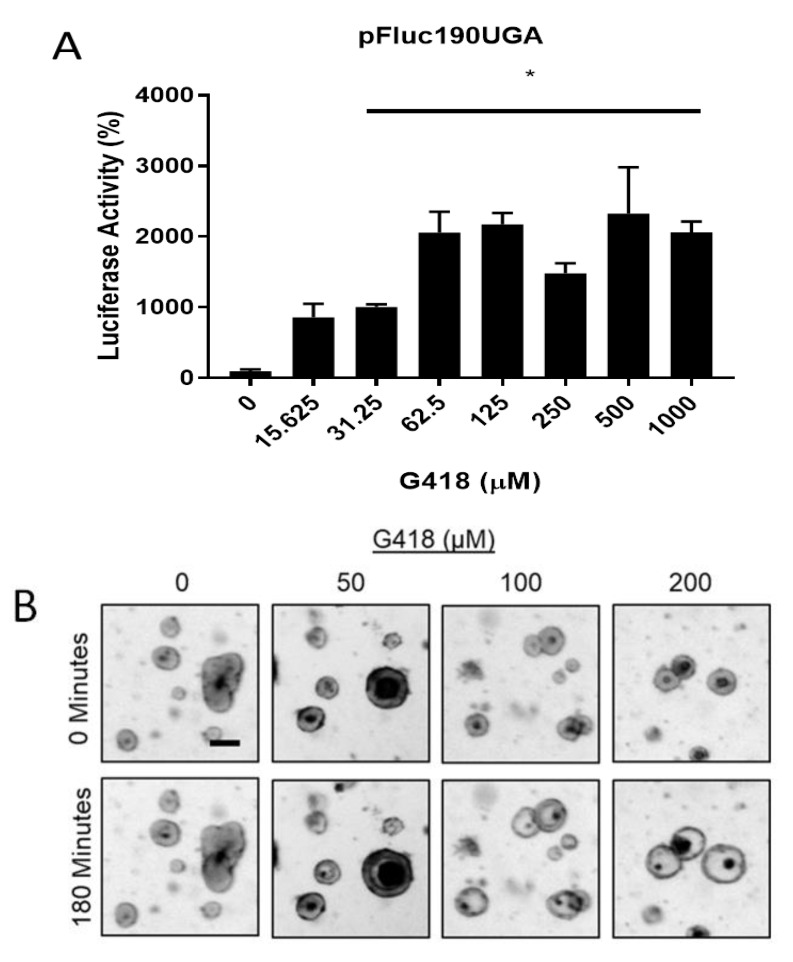

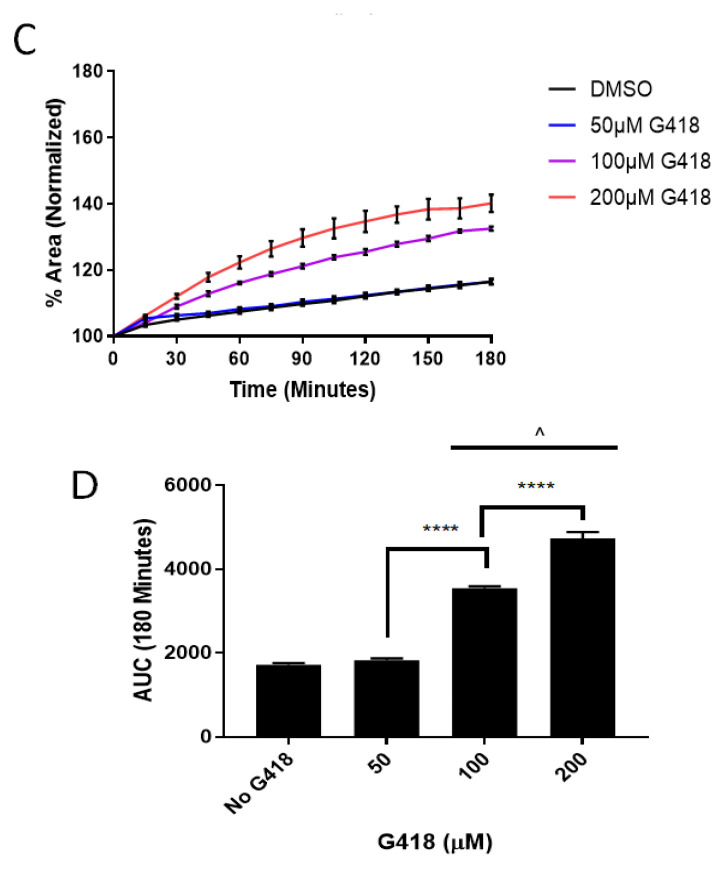
Readthrough with G418 in a cell based reporter system and G542X intestinal organoids (**A**). Firefly luciferase activity in 3T3 cells transfected with pFluc190UGA and treated with the indicated dose of G418. *n* = 3 wells per dose. * *p* < 0.0001 by one way ANOVA with post hoc Tukey test, ± SD. (**B**). Representative brightfield images of G542X intestinal organoids with indicated treatments at 0 and 180 min following stimulation with 10 µM forskolin. Scale bar is 100 µm. (**C**). FIS curves of intestinal organoids images every 15 min following forskolin stimulation. *n* = 3 wells per treatment group. (**D**). AUC measurements recorded from 1C. **** *p* < 0.0001, ^ *p* < 0.0001 vs. DMSO by one-way ANOVA with post hoc Tukey test.

Assays which measure ribosomal readthrough are commonly performed using transgenic cell-based reporters. Such reporters are highly sensitive to readthrough and have been utilized in high throughput screens to identify novel molecules [15]. However, only one of these molecules, PTC124, has undergone clinical trials for CF, and has not been found to have clinical benefit [37]. This suggests that reporter systems may not be optimal for detecting physiologically beneficial readthrough. We hypothesized that G542X intestinal organoids will more accurately recapitulate the effects of readthrough on G542X-Cftr in a physiologically relevant context. Therefore, we compared detection of readthrough with G542X intestinal organoid FIS to a cell-based readthrough reporter. To establish a readthrough reporter assay, we transfected mouse 3T3 fibroblast cells with pFluc190UGA [38] a plasmid which expresses firefly luciferase truncated by a UGA stop codon. Readthrough of the UGA stop codon allows detection of full-length luciferase. Transfected cells were treated with doses of G418 ranging from 0 to 1 mM for 24 h. We observed increases in luminescence ranging from 8.6 to 20.5 times greater than untreated cells for the G418-treated cells (Figure 1A), indicating dose-dependent readthrough of the UGA PTC. By comparison, G542X intestinal organoids were dosed with 50, 100, or 200 µM G418 for 24 h. *CFTR* function was then assessed by stimulating the organoids with 10 µM forskolin, and images of organoid FIS were captured by brightfield kinetic imaging (Figure 1B). The area under the curve (AUC) for each treatment was calculated to compare statistical significance between treatment groups. We observed robust FIS at 100 and 200 µM G418, but did not detect FIS at lower G418 doses (Figure 1C,D). An additional method to examine organoid swelling is by measurement of changes in organoid lumen size [39]. However, measurement of organoid lumen expansion did not increase the sensitivity of the FIS assay (Supplementary Figure S1A,B). Thus, G542X intestinal organoids required greater doses of G418 than pFluc190UGA to produce a detectable signal. These results suggest that G542X intestinal organoids may be less sensitive detectors of readthrough, but organoids more accurately model the physiological outcomes of readthrough in the context of CF nonsense mutations than cell-based reporter systems.

### 2.2. Inhibition of SMG-1 Synergizes with Readthrough to Restore CFTR Function

**Figure 2:**
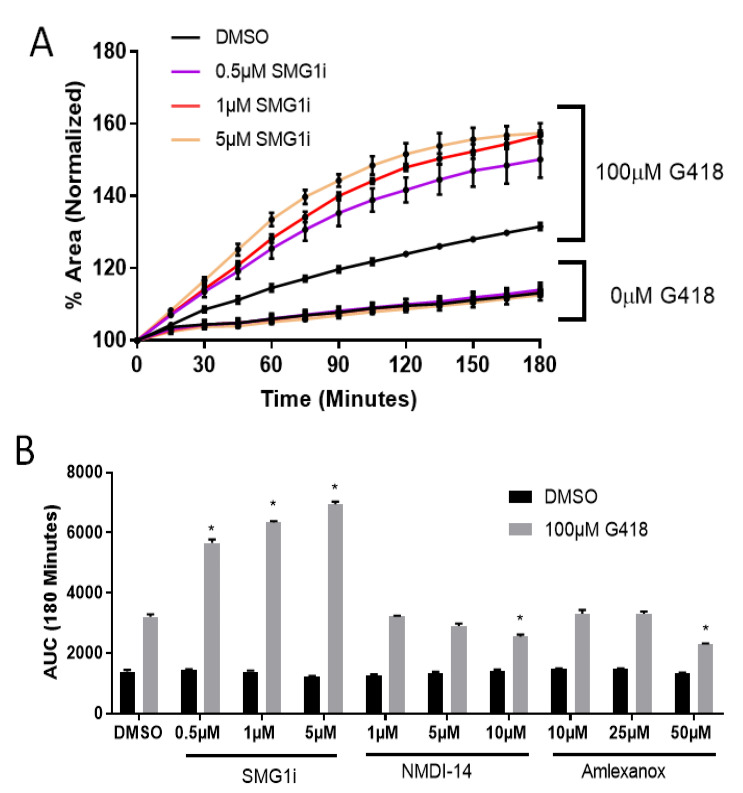

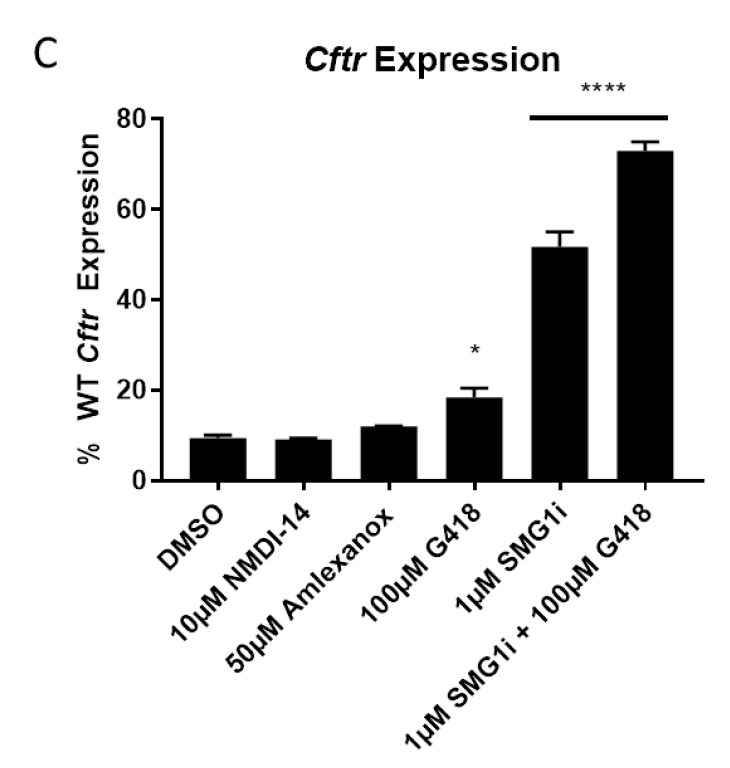
SMG1i, but not NMDI-14 or amlexanox, synergizes with G418 to improve *CFTR* function. (**A**). Normalized FIS curves of G542X intestinal organoids treated with indicated doses of SMG1i with or without 100 µM G418. *n* = 3. (**B**). AUC values for the indicated doses of SMG1i, NMDI-14, and amlexanox with or without 100 µM G418. * *p* < 0.001 vs. DMSO: DMSO by ANOVA with a post-hoc Tukey test. (**C**). *Cftr* mRNA expression in organoids treated for 24 h with the indicated doses of NMDI-14, amlexanox, G418, and SMG1i. Displayed as a percentage of WT *Cftr* expression. * *p* < 0.01, **** *p* < 0.0001 by 1 way ANOVA with post-hoc Tukey test. *n* = 3 experiments per group.

The G542X PTC is expressed in native Cftr in G542X mice, which makes G542X-Cftr mRNA sensitive to degradation by NMD. We sought to determine whether FIS mediated by readthrough of the G542X PTC could be increased by pharmacological inhibition of NMD. Intestinal organoids were incubated for 24 h with three small molecule inhibitors of NMD, SMG1i [21], NMDI-14 [20], and amlexanox [19] in combination with 100 µM G418. We observed robust synergy between G418 and SMG1i in our intestinal organoid system. FIS was significantly increased beyond G418 alone when several doses of SMG1i were combined with 100 µM G418 (Figure 2A,B). SMG1i did not allow FIS to occur in the absence of G418 at any tested dose, indicating that NMD inhibition alone is insufficient to restore *CFTR* function. Additionally, intestinal organoids from Cftr-null mutant S489X mice, which cannot produce functional CFTR after readthrough due to two neomycin genes that replace the rest of exon 11 [40,41], did not undergo FIS following treatment with G418 and SMG1i, indicating that improvements in FIS in G542X mice are due to inhibition of NMD (Supplementary Figure S2). By contrast, incubation with NMDI-14 did not provide any increase in FIS beyond 100 µM G418 alone at any tested dose (Figure 2B). We also did not observe synergy between amlexanox and G418 in G542X intestinal organoids at several doses (Figure 2B). Of note, the highest tested doses of amlexanox and NMDI-14 appeared to reduce swelling. This reduction in swelling appears independent of cellular toxicity, as intestinal organoids treated with highest tested doses of amlexanox and NMDI-14 did not have significantly greater cell death, as indicated by propidium iodide staining, than DMSO-treated organoids (Supplementary Figure S3).

To confirm that SMG1i improves FIS by increasing the quantity of *G542X-Cftr* mRNA, we performed RTqPCR on intestinal organoids treated with SMG1i and G418. We observed a 5.4-fold increase over DMSO treated organoids with 1 µM SMG1i treatment equating to 51.7% of WT expression, and a 7.6-fold increase when SMG1i was combined with 100 µM G418 equating to 72.9% of WT expression, indicating an increase in mRNA quantity (Figure 2C). Thus, readthrough occurs too rarely to produce sufficient protein for FIS even when mRNA quantity is greatly increased. NMDI-14 and amlexanox did not increase *G542X-Cftr* quantity, consistent with the results of our functional examination. Collectively, these results indicate that NMD inhibition by NMDI-14 and amlexanox are insufficient to improve CFTR function following readthrough, but SMG1i robustly inhibits NMD leading to readthrough and improved CFTR function.

### 2.3. Effectiveness of G418 Can Be Improved by NMD Inhibition

**Figure 3:**
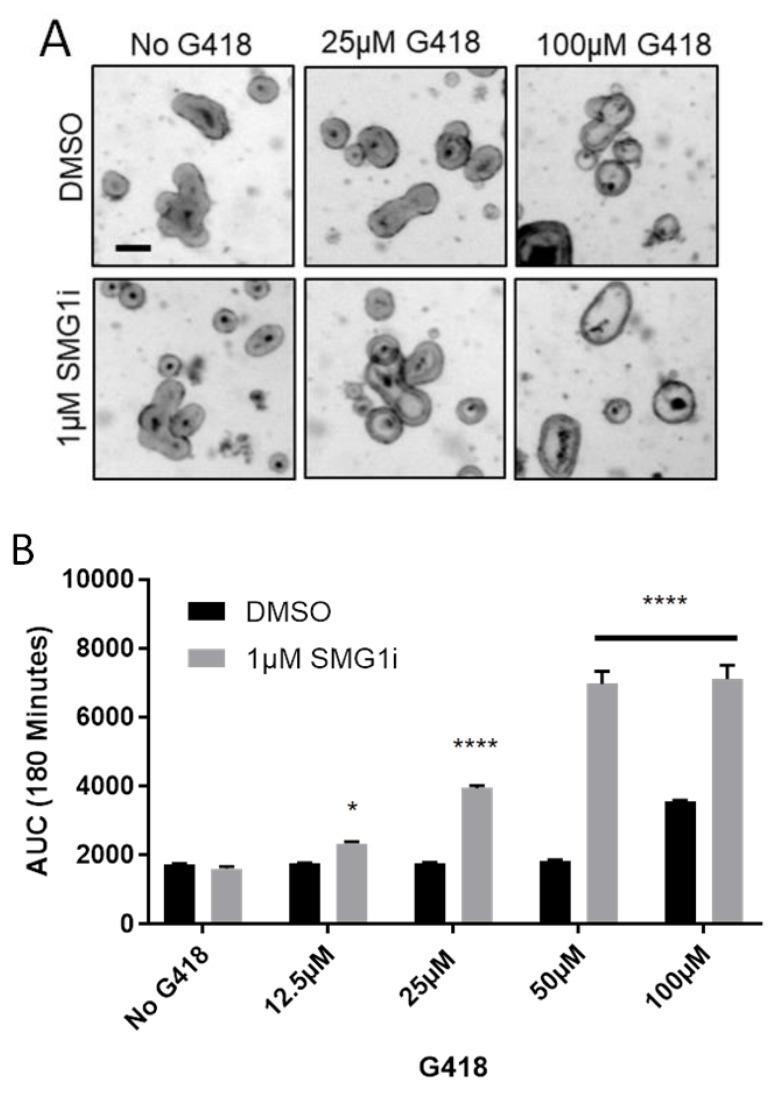
Low levels of readthrough are improved by inhibiting NMD. (**A**). Representative brightfield images of G542X intestinal organoids at 180 min following stimulation with 10 µM forskolin. Scale bar is 100 µm. (**B**). AUC measurements from swelling curves of G542X intestinal organoids treated with the indicated doses of G418 and/or 1 µM SMG1i. *n* = 3, * *p* < 0.01, **** *p* < 0.0001 vs. DMSO by two-way ANOVA with post-hoc Tukey test.

We next sought to determine if ineffective levels of readthrough could be improved with NMD inhibition. To this end, we treated G542X intestinal organoids with a range of G418 doses from 0 to 100 µM G418, and supplemented each dose with 1 µM SMG1i. Treatment with SMG1i was able to improve FIS when combined with several doses of G418 (Figure 3A,B). Notably, at 12.5, 25, and 50 µM G418 alone, no FIS was observed, but addition of 1 µM SMG1i was sufficient to significantly increase FIS. A combination of SMG1i and higher concentrations of G418 led to increased propidium iodide staining, indicating increased cell death (Supplementary Figure S4A). At high concentrations of G418 and SMG1i (200 µM G418 and 1 µM SMG1i), cellular toxicity was sufficient to cause a reduced FIS response (Supplementary Figure S4B). These results indicate that NMD inhibition by SMG1i increases the quantity of *G542X-Cftr* mRNA, leading to an increased quantity of functional *CFTR* following G418-mediated readthrough. However, there are toxicity concerns at higher concentrations.

### 2.4. SMG1i Improves Readthrough for Alternative Aminoglycosides to G418

**Figure 4:**
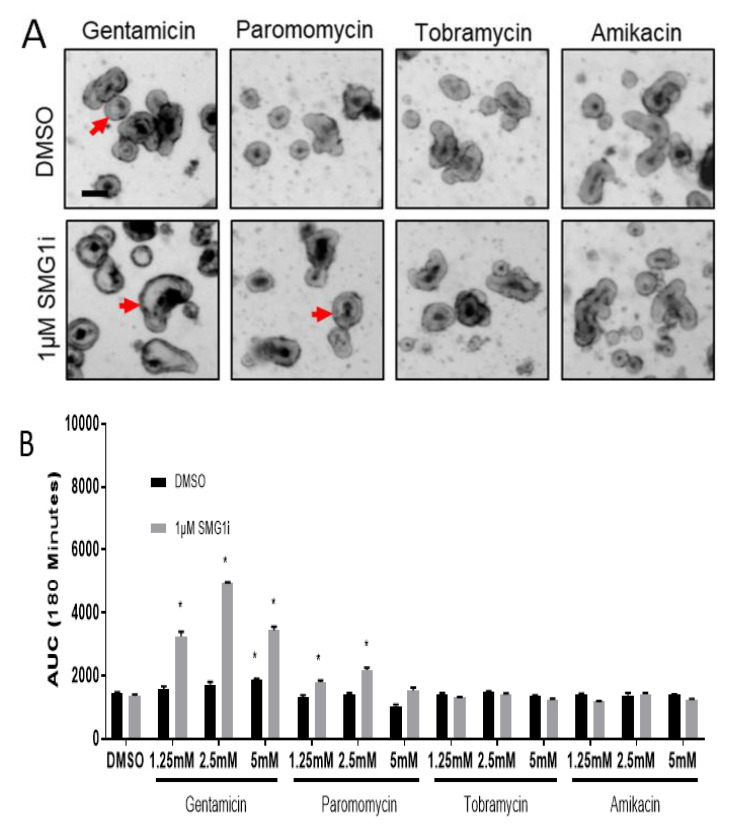

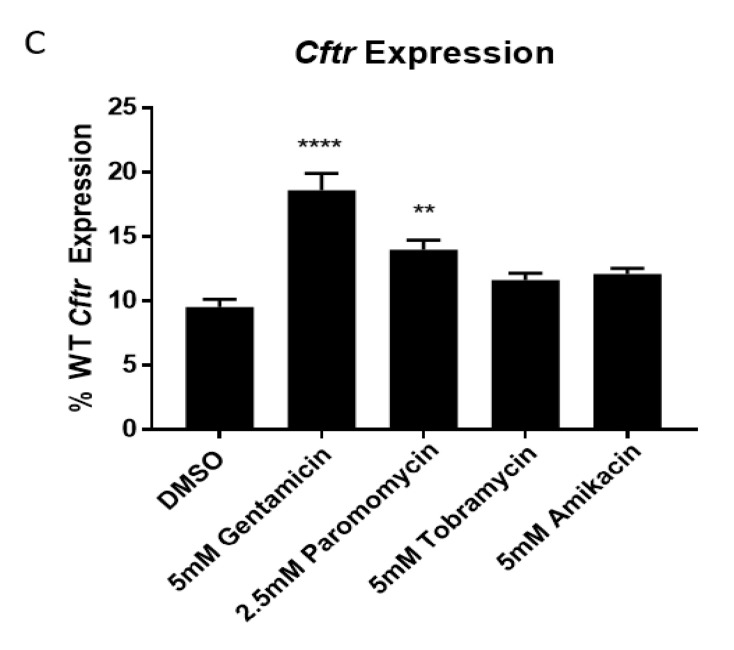
Gentamicin and paromomycin restore detectable levels of *CFTR* function. (**A**). Representative brightfield images of G542X intestinal organoids incubated with 2.5 mM of the indicated aminoglycosides 180 min after forskolin stimulation. Red arrows indicate expanded organoid lumens. Scale bar is 100 µm. (**B**). AUC measurements for four aminoglycosides at indicated doses. Each AUC is representative of 3–6 individual experiments. * *p* <0.05 (**C**). *CFTR* expression displayed as a percentage of WT Cftr expression. *n* = 3 wells per treatment group, ** *p* < 0.01; **** *p* < 0.0001 vs. untreated by one-way ANOVA with a Dunnett test.

We hypothesized that aminoglycosides which cause less readthrough than G418 could be more effective when paired with NMD inhibition. To examine readthrough caused by aminoglycoside alternatives to G418, we treated G542X intestinal organoids with gentamicin, amikacin, paromomycin, and tobramycin at three dilutions (5 mM, 2.5 mM, and 1.25 mM), with and without 1 µM SMG1i. We observed FIS only in organoids treated with gentamicin and in organoids treated with a combination of paromomycin and SMG1i, while tobramycin and amikacin did not allow FIS to occur (Figure 4A,B). Similar to G418, measurement of changes in organoid lumen size was not more sensitive to swelling than automated identification of total organoid area (Supplementary Figure S5). Of the four aminoglycosides tested, only paromomycin and gentamicin significantly increased *Cftr* expression, suggesting readthrough was occurring (Figure 4C), which is consistent with the observed increases in CFTR function. (Figure 4C). Furthermore, we examined readthrough caused by gentamicin, paromomycin, tobramycin, and amikacin using the readthrough reporter pFluc190UGA. We observed significant increases in luciferase expression for doses ranging from 0 to 5 mM of each aminoglycoside except for tobramycin, which did not significantly increase readthrough (Supplementary Figure S6A–D). These results indicate that combination treatment of paromomycin and gentamicin with SMG1i can produce small amounts of functional readthrough, with gentamicin producing the greatest readthrough of tested aminoglycosides.

### 2.5. Readthrough and NMD Inhibition Combine to Improve CFTR Function in Primary Trachea Cells

**Figure 5:**
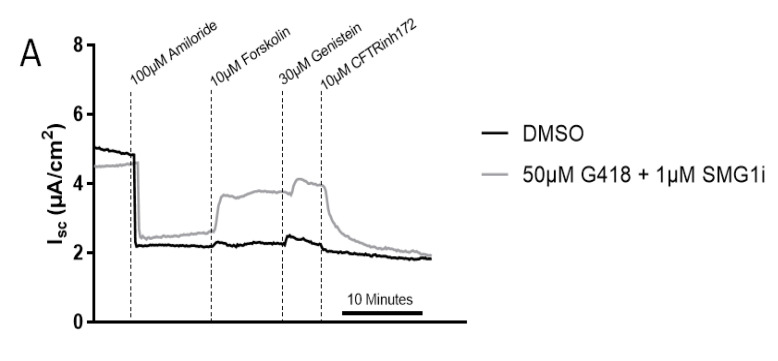

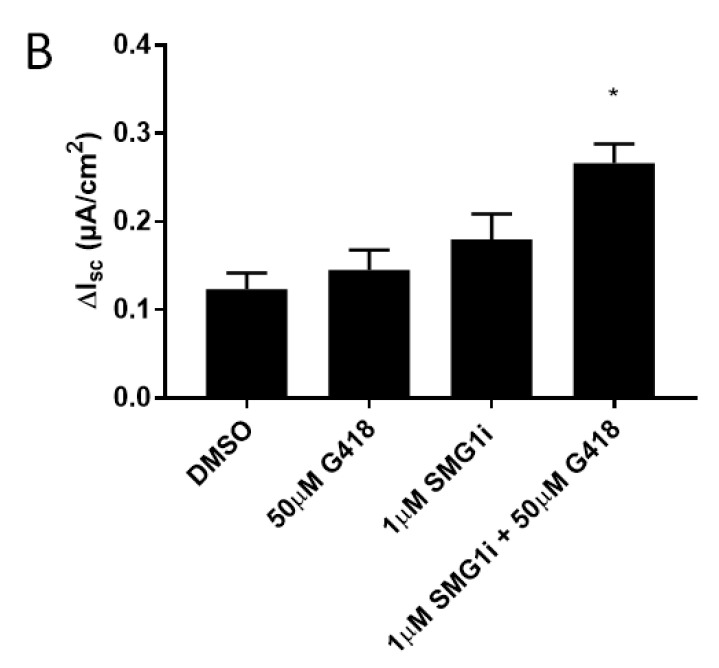
SMG1i synergizes with readthrough in trachea tissue. (**A**). Representative Isc traces of G542X trachea monolayers treated with DMSO or a combination of 50 µM G418, and 1 µM SMG1i for 48 h prior to treatment with compounds as indicated. Subsequent CFTR stimulation, potentiation and inhibition were achieved through the addition of forskolin, genistein, and CFTRinh172 (**B**). Changes in Isc after stimulation with 10 µM forskolin for the indicated treatment groups. *n* = 3–11, * *p* < 0.05.

To examine synergy of SMG1i with readthrough agents in a non-intestinal tissue type, G542X tracheal and nasal epithelial cells isolated from G542X mice were grown as air-liquid interface cultures and treated with 50 µM G418 for 48–72 h, with or without 1 µM SMG1i. CFTR-dependent short-circuit current (I_sc_) was increased by G418 and SMG1i co-treatment; whereas neither compound alone increased CFTR function (Figure 5A,B). These experiments indicate synergy between readthrough agents and NMD inhibition in airway tissue. For a detailed description of experimental parameters, please refer to Section 4.7.

### 2.6. Non-Aminoglycoside Compounds Do Not Cause Sufficient Readthrough to Restore CFTR Function

**Figure 6:**
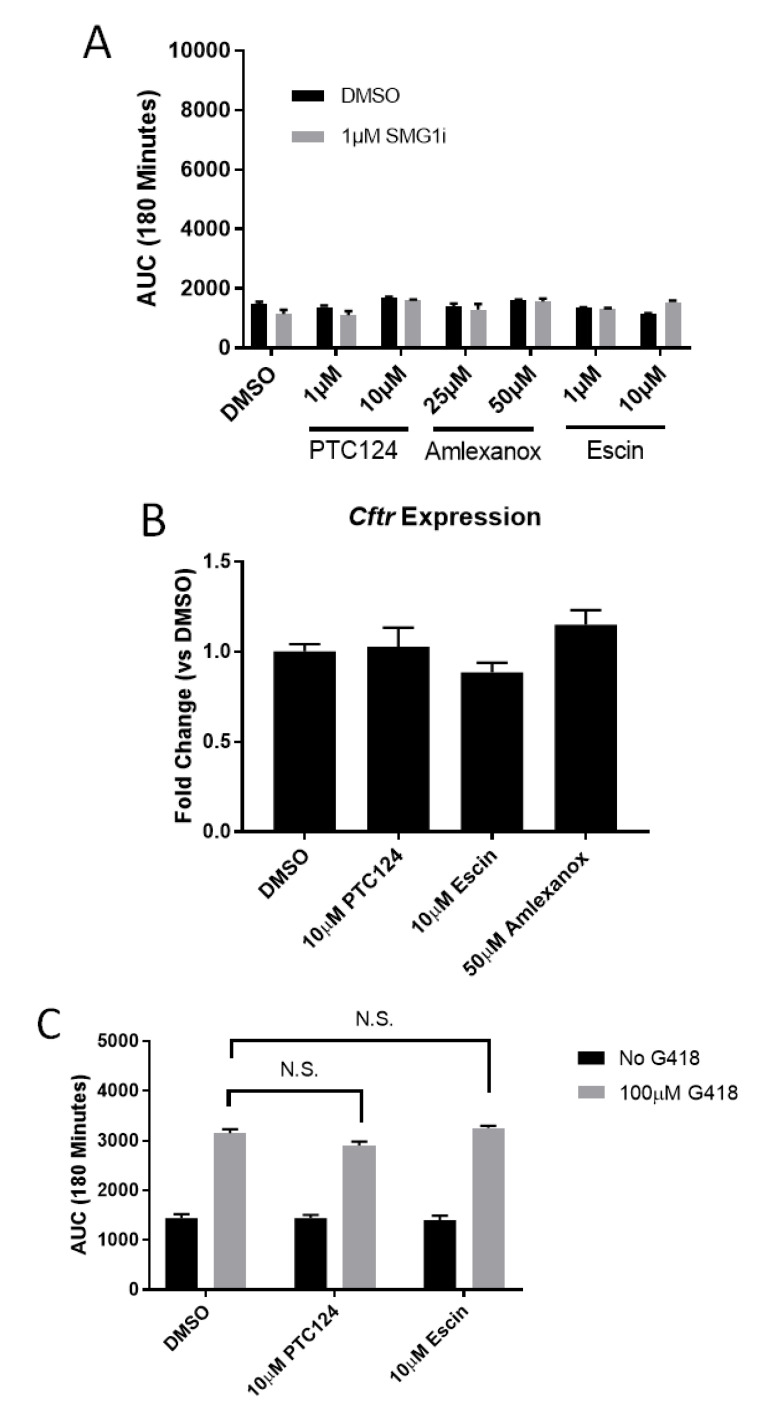
Non-aminoglycoside readthrough agents do not restore *CFTR* function in G542X intestinal organoids. (**A**). AUC measurements from G542X intestinal organoids treated with the indicated doses of PTC124, amlexanox, and escin with or without SMG1i. *n* = 3. (**B**). Cftr expression displayed as fold change vs. DMSO after 24 h incubation with the indicated com-pounds. (**C**). AUC measurements from intestinal organoids incubated for 24 h with the in-dicated compounds. *n* = 3–6 wells, analyzed using two way ANOVA with post-hoc Tukey test. All G418-treated groups are significantly increased over no G418 groups.

Next, we sought to examine synergy of non-aminoglycoside readthrough agents with SMG1i. We examined FIS in G542X intestinal organoids following a 24 h incubation with PTC124, escin, and amlexanox in conjunction with SMG1i treatment. The concentration range of each drug included doses used in other publications [15] as well as the highest nontoxic concentration observed in organoids. No tested dose of any compound allowed FIS to occur, with or without combination treatment with SMG1i (Figure 6A). The highest tested doses of PTC124 and escin did not increase *Cftr* expression, suggesting minimal readthrough (Figure 6B). To test for possible synergy between non-aminoglycoside and aminoglycoside readthrough agents, we examined PTC124, escin, or amlexanox in combination with 100 µM G418, but found no evidence of synergy as the addition of the non-aminoglycoside did not increase FIS response to G418 (Figure 2A and Figure 6C). Finally, we utilized the readthrough reporter system pFluc190UGA with each non-aminoglycoside readthrough agent. For amlexanox and escin, we observed a significant increase in luminescence but very small compared to aminoglycosides (Supplementary Figure S7A,B). We did not use PTC124 in this system as PTC124 has been reported to stabilize firefly luciferase in this system, providing a false positive [38]. Collectively, these results indicate that non-aminoglycoside readthrough agents do not cause sufficient readthrough to restore functional levels of CFTR, even when supplemented with NMD inhibition.

### 2.7. G542X-CFTR Trafficking Can Be Improved by a CFTR Corrector Following Readthrough with G418 and Gentamicin

**Figure 7:**
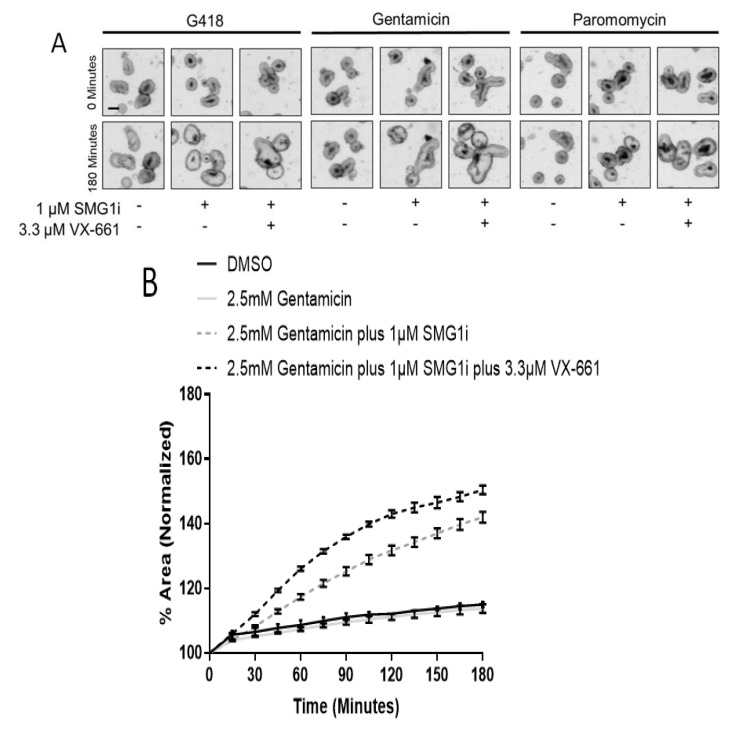

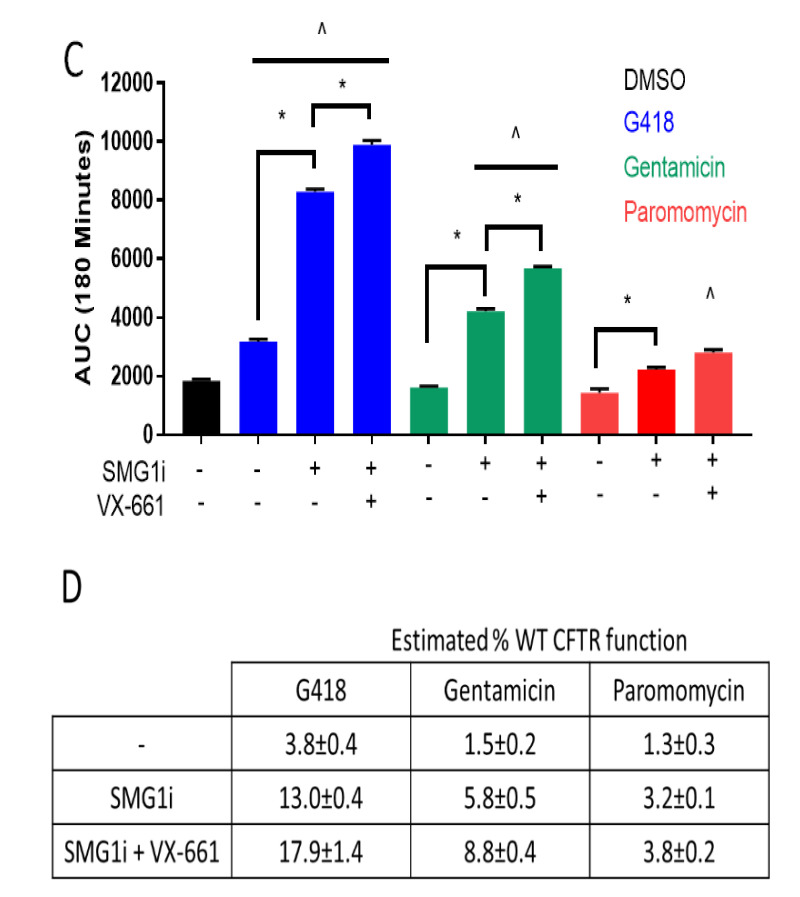
Folding of readthrough-facilitated CFTR can be improved with VX-661. (**A**). Representative brightfield images of G542X intestinal organoids treated with 100 µM G418, 2.5 mM gentamicin, or 2.5 mM paromomycin and indicated combinations of 1 µM SMG1i and/or 3.3 µM VX-661 at 0 min and 180 min following treatment with 10 µM forskolin. Scale bar is 100 µm. (**B**). Example FIS curves of G542X intestinal organoids treated with indicated doses. *n* = 3 wells per treatment group. (**C**). AUC measurements from intestinal organoids treated with the indicated doses of compounds as in 6A. * *p* < 0.0001 between indicated treatment groups, ^ *p* < 0.0001 from DMSO by one-way ANOVA with post-hoc Tukey test. (**D**). Estimated percent of wild-type CFTR function comparing the initial rate of FIS of each group to wild-type ± SD. G542X organoids with no NMD, readthrough or corrector agents had an estimated 1.3 ± 0.7% of wild-type CFTR function. The initial rate was calculated from the linear phase of FIS swelling which was 10 minutes for wild-type and 45 min for G542X.

Readthrough frequently leads to the insertion of a non-optimal amino acid in place of the PTC [42]. This non-optimal amino acid can cause dysfunctional CFTR trafficking [42]. Several small molecules; known as correctors, are available for correcting abnormal CFTR trafficking, which is common to mutant CFTR [28,43]. To examine modification of protein trafficking following readthrough, we incubated intestinal organoids with the corrector VX-661 along with G418, gentamicin, or paromomycin with SMG1i. We found that VX-661 allowed greater FIS than readthrough agents and SMG1i alone (Figure 7A–D), indicating correction of aberrant CFTR trafficking and increased function of CFTR at the apical membrane. Assuming the initial rate of a FIS curve estimates maximal CFTR function, we compared the mean initial rate of FIS of each group to the wild-type mean initial rate of FIS to estimate a percentage of wild-type CFTR function that was corrected (Figure 7D). We did not observe FIS in G542X intestinal organoids treated with VX-661, confirming that VX-661 does not allow FIS to occur independent of CFTR function (Supplementary Figure S8). These data indicate that the trafficking of murine G542X-CFTR can be improved by administration of correctors after readthrough and NMD inhibition.

## 3. Discussion

A number of heritable diseases including CF, Duchenne Muscular Dystrophy (DMD) [10,16] and Spinal Muscular Atrophy (SMA) [44] can arise due to nonsense mutations. Considering the prevalence of diseases caused by nonsense mutations, a therapy which can restore function to genes ablated by nonsense mutations is highly desirable. Although a safe and highly effective readthrough agent has yet to be identified, currently existing readthrough agents may be more effective if administered alongside inhibitors of NMD. Here, we identify several currently available readthrough agents which are more effective in combination with a pharmacological inhibitor of SMG-1, a critical component of NMD, using functional assays of murine G542X-CFTR. Furthermore, we highlight the benefits of an intestinal organoid system of CF nonsense mutations, which allows examination of readthrough of PTCs in *Cftr* which is sensitive to NMD and expressed by a native promoter.

We examined three NMD-inhibiting compounds in this study, NMDI-14 [20], amlexanox, and SMG1i [21]. We did not find NMDI-14 or amlexanox to be effective for improving FIS mediated by G418-facilitated readthrough. However, we found synergy between SMG1i and several readthrough agents to improve G542X-CFTR function. We also found SMG1i to have a degree of toxicity, particularly when combined with higher doses of aminoglycosides. Toxicity caused by SMG1i is unsurprising, as SMG-1 is active in a number of cellular processes outside of NMD [45,46], and interfering with SMG-1 activity likely has deleterious effects on cell health. This raises the possibility that toxicity from SMG1i by itself or in combination with readthrough therapy would preclude SMG1i from use in a clinical setting and a thorough examination of in vivo SMG1i toxicity is necessary. Regardless of clinical efficacy, SMG1i serves as a proof of concept that a compound can inhibit NMD to a degree which is sufficient to improve G542X-CFTR function after readthrough. Considering that the toxicity of aminoglycosides at the doses required for readthrough precludes their use in a clinical setting, supplementation with NMD inhibitors may represent a strategy for increasing aminoglycoside-facilitated readthrough with non-toxic aminoglycoside doses.

In our intestinal organoid experiments, we observed varying readthrough efficacy by several different aminoglycosides. Consistent with previous research, G418 provided the strongest readthrough effect [47] and synergized effectively with SMG1i. We also observed this effect in air-liquid interface cultures of primary airway cells from G542X mice. These observations are consistent with Valley and colleagues, who observed synergy between G418 and SMG-1 inhibition in a CRISPR-edited human bronchial epithelial (HBE) cell line [48]. Gentamicin, though not as effective as G418, did allow a small degree of FIS. When combined with SMG1i, gentamicin facilitated a robust FIS response. Further improvement of *CFTR* function was observed by treating the intestinal organoids with the CFTR trafficking corrector VX-661, consistent with what was observed by Xue and colleagues, who observed that the corrector VX-809 [43] was sufficient to improve folding of human G542X-CFTR following readthrough [42]. We consider these findings significant, as gentamicin is the only aminoglycoside which has been utilized in clinical trials to treat nonsense mutations. However, gentamicin was dropped from clinical trials due to causing ototoxicity and nephrotoxicity. The possibility of combining gentamicin with an NMD inhibitor and CFTR corrector in a clinical setting may improve the efficacy of gentamicin to a clinically relevant level. With inhibition of NMD, readthrough by gentamicin and the CFTR modulator VX-661, G542X intestinal organoids had an estimated 8.8% of wild-type CFTR function which may be enough to ameliorate certain CF disease manifestations [49]. A similar strategy was applied to the W1282X mutation in human derived nasal cells resulting in some recovery of functional CFTR [50].

Novel interventions are being developed which may further improve CFTR function following readthrough. Recently, a compound named PTI-428 has been identified which selectively increases *CFTR* expression has been identified by high-throughput screening [51]. PTI-428 represents a novel class of CFTR modulators which are termed “amplifiers”, which increase *CFTR* gene transcription. Increasing *CFTR* expression in the context of a *CFTR* nonsense mutation may further improve the quantity of mutant mRNA which can be acted on by readthrough agents.

The non-aminoglycoside readthrough agents escin and PTC124 did not cause enough readthrough to restore CFTR function or to increase *Cftr* expression. There is abundant published research which suggests that each molecule is able to cause ribosomal readthrough of PTCs [15,30]. However, many of these experiments were performed using cell-based readthrough reporters which rely on overexpressed transgenic constructs to detect readthrough. As we have shown here, transgenic systems can be sensitive to low levels of readthrough which may be insufficient to translate to physiological functionality. Readthrough by PTC124 has been observed in vivo using mouse models of CF which overexpress human *CFTR* carrying a nonsense mutation [9]. Despite success in this model, PTC124 was ineffective for human nonsense-carrying intestinal organoids [52], and has not improved clinical outcomes in patients with nonsense mutations in *CFTR* [37,53]. However, PTC124 has been sufficient to restore function to the dystrophin gene in a mouse model of Duchenne muscular dystrophy [14]. Therefore, despite the ineffectiveness in restoring *G542X-CFTR*, PTC124 may be sufficient to restore function to other genes which carry PTCs. Further study of these compounds with NMD inhibition in human CF primary cells, such as nasal epithelia and intestinal organoids, may provide further insight on their apparent lack of efficacy in CF.

In summary, primary cells from a CF nonsense mutation mouse model can effectively assess readthrough, synergy with NMD inhibition, and the resulting CFTR function. Considerable effort is currently being put forth to identify novel readthrough agents which can be both safe and highly effective in a clinical setting. However, even the most effective readthrough agent may still not restore enough gene function to reverse disease manifestations. Therefore, co-treatment with NMD inhibition may be the only viable option for sufficiently restoring gene function. The CF G542X intestinal organoid model represents a robust model for analyzing the effectiveness of newly identified compounds prior to in vivo treatment studies. Here, we demonstrate that treatments for nonsense mutations which combine NMD inhibition with readthrough, and then further interventions to modify protein function may be a more effective therapeutic option than readthrough alone.

## 4. Materials and Methods

### 4.1. Mice

The creation of the G542X mouse model was previously described [29]. Mice homozygous for these mutations were created by breeding heterozygous males and females. Genotyping was completed by PCR analysis using DNA extracts from ear biopsies. To detect the G542X allele (319 bp) primers P1 (5′-ACAAGACAACACAGTTCTCT-3′) and P2 (5′-TCCATGCACCATAACAACAAGT-3′) were used. To detect the wild-type (WT) allele (319 bp) P2 and P3 (5′-ACAAGACAACACAGTTCTTG-3′) were used in a separate reaction. PCR reactions were completed for 40 cycles of 95 °C for 30 s, 58 °C for 30 s and 72 °C for 30 s and products were run out on 2% agarose gels. All mice were allowed unrestricted access to water and solid chow (Teklad 7960; Envigo, Indianopolis, IN, USA). All animals were maintained on a 12 h light, 12 h dark schedule at a mean ambient temperature of 22 °C and were housed in standard polysulfone microisolator cages in ventilated units with corncob bedding. The Institutional Animal Care and Use Committee of Case Western Reserve University approved all animal protocols (#2014-0064; April 2017).

### 4.2. Compounds

Stocks of aminoglycoside antibiotics (All purchased from Sigma-Aldrich, St. Louis, MO, USA) were dissolved in sterile H_2_O at a 100 mg/mL concentration, and stored at 4 °C. PTC124 (3-[5-(2-Fluorophenyl)-1,2,4-oxadiazol-3-yl]benzoic acid, MedChemExpress, Cat. #HY-14832), escin (Sigma-Aldrich, Cat. #E1378), VX-661 (MedChemExpress, Monmouth Junction, NJ, USA Cat. #HY-15448), NMDI-14 (Ethyl 2-(((6,7-dimethyl-3-oxo-1,2,3,4-tetrahydro-2-quinoxalinyl)acetyl)amino)-4,5-dimethyl-3-thiophenecarboxylate, EMD Millipore, Burlington, MA, USA, Cat. #530838), and amlexanox (MedChemExpress Cat. #HY-B0713) were dissolved in dimethylsulphoxide (DMSO). SMG1i (2-chloro-N,N-diethyl-5-((4-(2-(4-(3-methylureido)phenyl)pyridin-4-yl)pyrimidin-2-yl)amino)benzenesulfonamide) was received from Dr. Robert Bridges from Rosalind Franklin University of Medicine and Science through the CFFT compound distribution program, and was dissolved in DMSO. Working solutions were all ≤0.1% DMSO, with the exception of NMDI-14, which was 1% DMSO. The increased DMSO did not prevent G418-facilitated FIS (Supplementary Figure S9). Stocks of forskolin (Sigma-Aldrich, Cat. #F6886) were dissolved in 100% EtOH and stored at −20 °C.

### 4.3. Crypt Harvest and Intestinal Organoid Culture

Intestinal organoids were cultured similar to previously described methods [29]. Mice were sacrificed by CO_2_ asphyxiation, and 20 cm of intestine measured from the stomach were removed. Fecal matter was flushed from the intestine with Ca^2++^ and Mg^2++^-free PBS, and the intestine was flayed using dissecting scissors. The villi were scraped from the small intestine using a microscope slide, and the intestine was cut into ~1 cm segments, which were suspended in 2mM EDTA in Ca^2++^ and Mg^2++^-free PBS. The intestinal segments were incubated on a shaker for 30 min at room temperature. The segments were then vortexed at for 10 s, allowed to settle, and then the supernatant was removed and stored in a 10 cm dish. This process was repeated until four supernatant fractions were produced. The fractions were inspected under a microscope, and the fraction which was most enriched for crypts was passed through a 70 µm cell strainer. The crypts were pelleted at 1000× *g* for 10 min, then resuspended in 1:1 mixture of Intesticult Organoid Growth Media (OGM; Stemcell Technologies, Cambridge, MA, USA) and MatriGel (Corning, Corning, NY, USA) at a concentration of 10 crypts/µL. The organoids were seeded to 12-well plates, with 70 µL Matrigel:OGM added to each well in 4–5 droplets. The plate was placed in a 37 °C/5% CO_2_ incubator for 15 min to allow the MatriGel to harden. The MatriGel domes were then immersed in 1mL OGM and returned to the 37 °C/5% CO_2_ incubator. OGM was changed every 3–4 days, and the organoids were passaged once every 5–7 days.

### 4.4. Forskolin-Induced Swelling Assay

Organoid swelling experiments were carried out similar to previously described methods [29,31], with small modifications. Organoids were collected into a 15 mL conical tube using Ca^2++^ and Mg^2++^ free PBS and pelleted at 500× *g*. The organoids were resuspended in 1 mL PBS and dissociated by vigorously resuspending the organoids 10–15 times with a P1000 pipette tip covered with a P10 pipette tip. The dissociated organoids were pelleted and resuspended in 1:1 OGM:MatriGel. The organoids were diluted to a concentration of 30–50 organoids per 5 µL MatriGel droplet, and plated to 48-well plates with 5 µL of MatriGel per well. The plate was then incubated for 15 min in a 37 °C/5% CO_2_ incubator to harden the MatriGel. Immediately following hardening of MatriGel, indicated dilutions of readthrough agents and NMD inhibitors were added along with 200 µL OGM. To assess NMD inhibition, we utilized 1 µM SMG1i for the majority of experiments. 5 µM SMG1i had allowed the largest FIS response of any tested doses when combined with G418, however, we selected 1 µM SMG1i as the optimal dose due to concerns about toxicity. Twenty four hours following addition of readthrough agents, 200 µL OGM containing 20 µM forskolin was added to each well, creating a 10 µM final concentration. Kinetic brightfield images of FIS were acquired under live cell conditions with a Lionheart FX automated microscope (Biotek Instruments, Winooski, VT, USA). FIS was quantified using Gen5 Prime software (Biotek Instruments, Winooski, VT, USA) by normalizing changes in organoid sum area to the sum area in the initial image. Area under the curve (AUC) was calculated using Prism (Graphpad, San Diego, CA, USA). Expansion of the organoid lumen was measured by marking the organoid lumen at 0 min and 180 min using the polygon selection tool in ImageJ (NIH, Rockville, MD, USA). Lumen expansion was quantified as the percent change in lumen area between the 0 min and 180 min. Ten organoids were measured per treatment group.

### 4.5. Toxicity Assessment

Intestinal organoids were passaged to 96 well plates and treated with indicated compounds for 24 h. The intestinal organoids were then stained with 10 µg/mL Hoechst and 1µg/mL propidium iodide for 30 min. Images were recorded using RFP and DAPI channels, and total RFP and DAPI intensity per well were calculated using Gen5 Prime software. RFP intensity was divided by DAPI intensity to normalize propidium iodide signal to the quantity of organoids.

### 4.6. Murine Primary Airway Cell Culture

Trachea or nasal epithelium was isolated from mice, cleaned, digested with pronase/DNase and followed by a brief treatment with accutase. Cells are co-cultured with irradiated fibroblasts and allowed to proliferate in a media containing a Rho-kinase inhibitor [54,55]. After proliferation, cells were seeded into 12-well plate inserts at a density of ~2 × 10^5^ cells per filter. Cells were switched to a media that promotes differentiation and grown in an air-liquid interface (ALI). After three weeks at ALI, the establishment of electrically tight cultures were determined by Ussing chamber measurements at which time assessment of *CFTR* function by short-circuit current measurement was completed.

### 4.7. Assessment of CFTR Function by Short-Circuit Current Measurement

The epithelial monolayers were bathed with symmetrical Krebs bicarbonate ringers solution, and maintained under short-circuit conditions. *CFTR* function was assessed as previously described [56]. Briefly, after stabilization of baseline, following inhibitors and activators of I_sc_ were sequentially added: sodium (Na^+^)-channel blocker Amiloride (100 μM) to inhibit apical epithelial Na^+^ channel (ENaC); cAMP agonists Forskolin (10 μM) and 3-isobutyl-1-methylxanthine (IBMX 100 μM) to activate the transepithelial cAMP-dependent current (including Cl^−^ transport through *CFTR* channels); genistein (30 μM) to potentiate *CFTR*; and *CFTR* inhibitor CFTRinh172 (10 μM) to specifically inhibit *CFTR*. Data were acquired with the software Acquire and Analyze version 2.3.159 (Physiologic Instruments). *CFTR* specific function in the cells was calculated as change in I_sc_ (ΔI_sc_) defined as the difference between the sustained phase of the current response before and after stimulation with forskolin.

### 4.8. Expression Analysis

To examine Cftr expression, G542X intestinal organoids were passaged into 24-well plates, with 35 µL of MatriGel per well. One well was considered to be a single experiment. The organoids were grown for five days to the point of being significantly budded, and were then treated for 24 h with indicated compounds diluted in 500 µL OGM. The organoids were then lysed using a QiaShredder Cell and Tissue Homogenizer Kit (Qiagen, Germantown, MD, USA, cat. #79654). RNA was harvested using an RNeasy Mini Kit (Qiagen, cat. #74104). 250 ng of RNA was reverse transcribed into cDNA using a QScript cDNA synthesis kit (VWR, Radnor, PA, USA, cat. #101414-098). Cftr expression was examined using a TaqMan expression assay which used primers recognizing exons 17 and 18 on Cftr (Mm00445197; ThermoFisher Scientific,). RTqPCR was performed on a StepOne PCR system (Applied Biosystems) to examine Cftr expression, with ß-actin serving as the endogenous control. The average of each value was expressed as the fold change difference in Cftr expression from DMSO-treated organoids.

### 4.9. Readthrough Reporter

Mouse 3T3 fibroblast cells (a kind gift from Mitchell Drumm) were grown in DMEM with 10% fetal calf serum and 1% penicillin/streptomycin. The cells were split to 96-well plates, with 10,000 cells per well. The following day, the cells were transfected with 100 ng per well of pFluc190UGA (a kind gift from James Inglese; Addgene plasmid #41046) [38] using Lipofectamine 2000 (ThermoFisher Scientific, Waltham, MA, USA, Cat. #11668030). 48 h following transfection, the cells were treated with indicated doses of readthrough agents for 24 h. Readthrough was assessed by quantifying luminescence of firefly luciferase using a Luciferase Assay System (Promega, Madison, WI, USA, Cat. # E4550). Luminescence was recorded using a GloMax Navigator Microplate Luminometer (Promega, cat. # GM2000). Luciferase activity in the experimental groups are displayed as percentage signal beyond DMSO-treated cells, similar to Auld et al. [38].

### 4.10. Statistical Analysis

All statistical tests were calculated using Prism. Specific tests were calculated for each experiment, and are indicated in the figure legends. All error bars are displayed as ± SEM unless otherwise indicated.

## Data Availability

Data is contained within this article or supplementary material.

## Supplementary Materials

Supplementary materials can be found at https://www.mdpi.com/1422-0067/22/1/344/s1.
