# Supplementary material for: Synergy between Readthrough and Nonsense Mediated Decay Inhibition in a Murine Model of Cystic Fibrosis Nonsense Mutations
Source: Int J Mol Sci. 2020 Dec 31;22(1):344. doi: 10.3390/ijms22010344 (PMC7794695; doi:10.3390/ijms22010344)
Supplement: Supplementary file 1 [file ijms-22-00344-s001.pdf]

**Supplementary Figures**

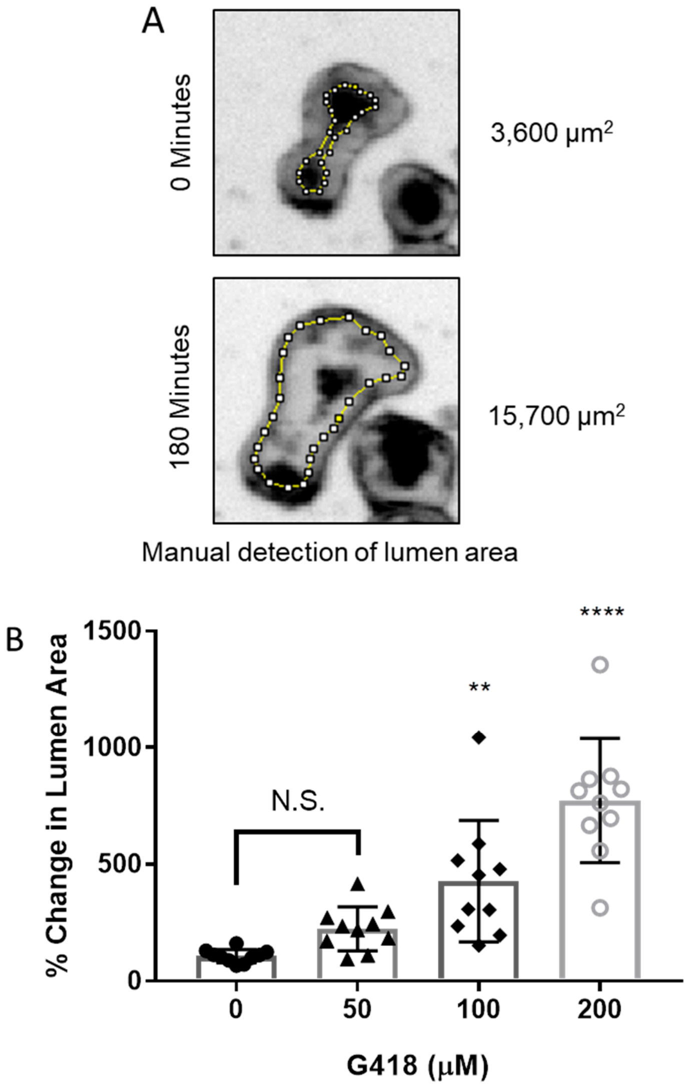


Supplementary Figure 1. Detection of FIS by measurement of the intestinal organoid lumen. A. Images of intestinal organoid lumen measurements at 0 and 180 minutes. B. Percent change in lumen area at 180 minutes. *n*=10 organoids per group. *p***=0.0036, *p*****<0.0001 compared to untreated control by one-way ANOVA with post-hoc Tukey test.

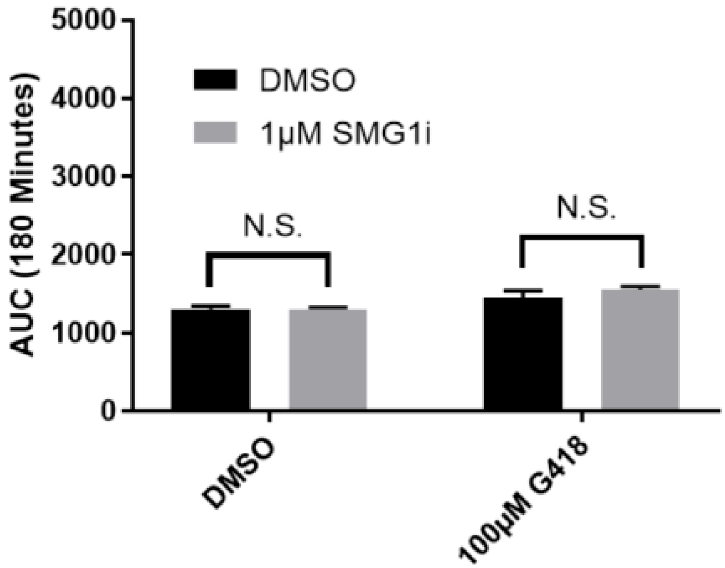


Supplementary Figure 2. FIS increases caused by SMG1i do not occur independently of NMD inhibition. S489X intestinal organoids were stimulated with 10μM forskolin following incubation with the indicated compounds. Data were analyzed using a two-way ANOVA with post-hoc Tukey test. *n*=3.

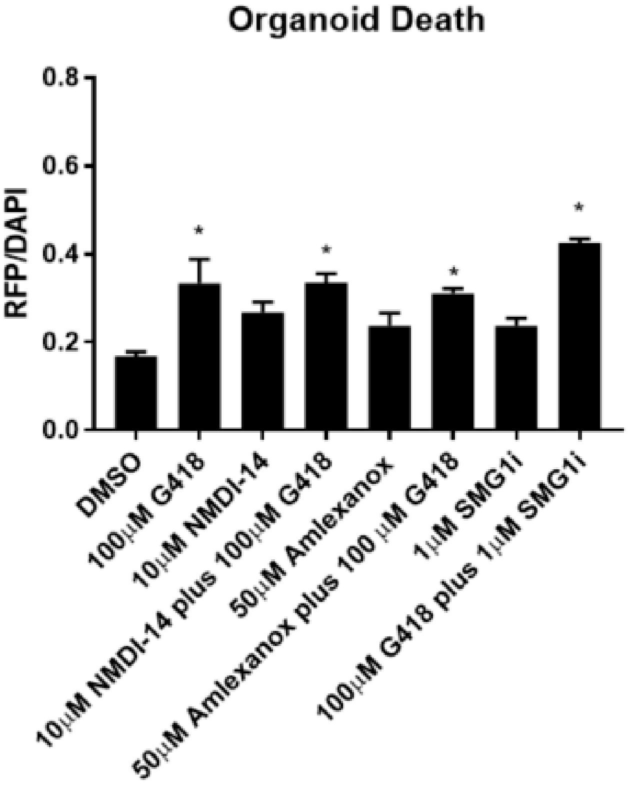


Supplementary Figure 3. Assessment of NMD inhibitor toxicity. Intestinal organoids were treated for 24 hours with the indicated compounds. $p^*<0.005$ vs DMSO by one way ANOVA with a post-hoc Tukey test. $n$=4-8 wells per treatment group.

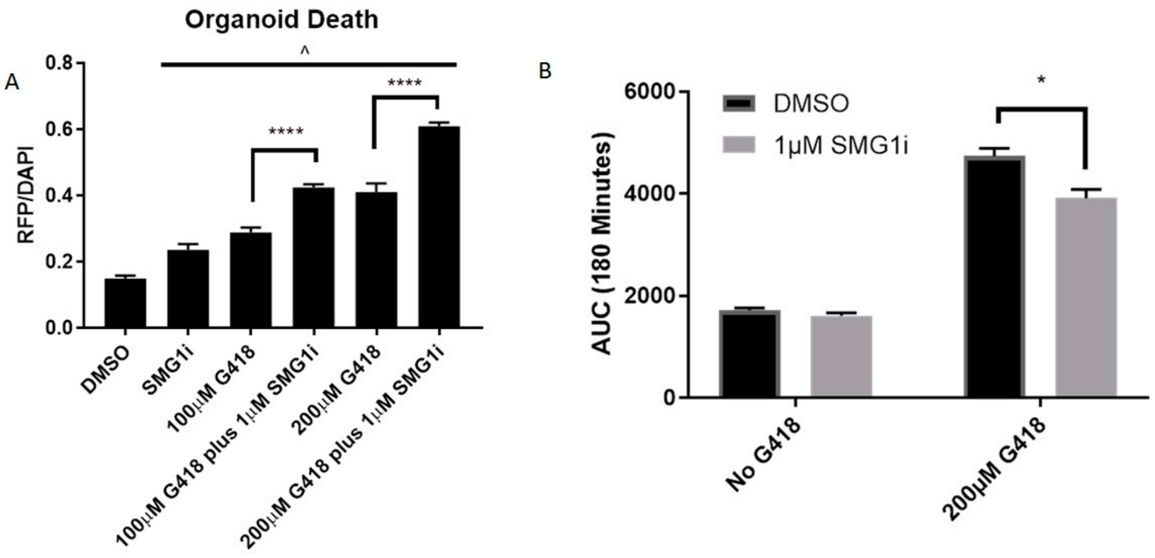


Supplementary Figure 4. SMG1i toxicity impedes FIS when combined with high doses of G418. A. RFP/DAPI values for intestinal organoids treated for 24 hours with the indicated compounds. *n*=4 wells per treatment, *p*****<0.0001 between indicated groups, *p*^<0.05 vs DMSO by one way ANOVA with post-hoc Tukey test. B. AUC values for intestinal organoids treated with the indicated compounds for 24 hours. *p**<0.05 between indicated groups by two-way ANOVA with post-hoc Tukey test. *n*=3.

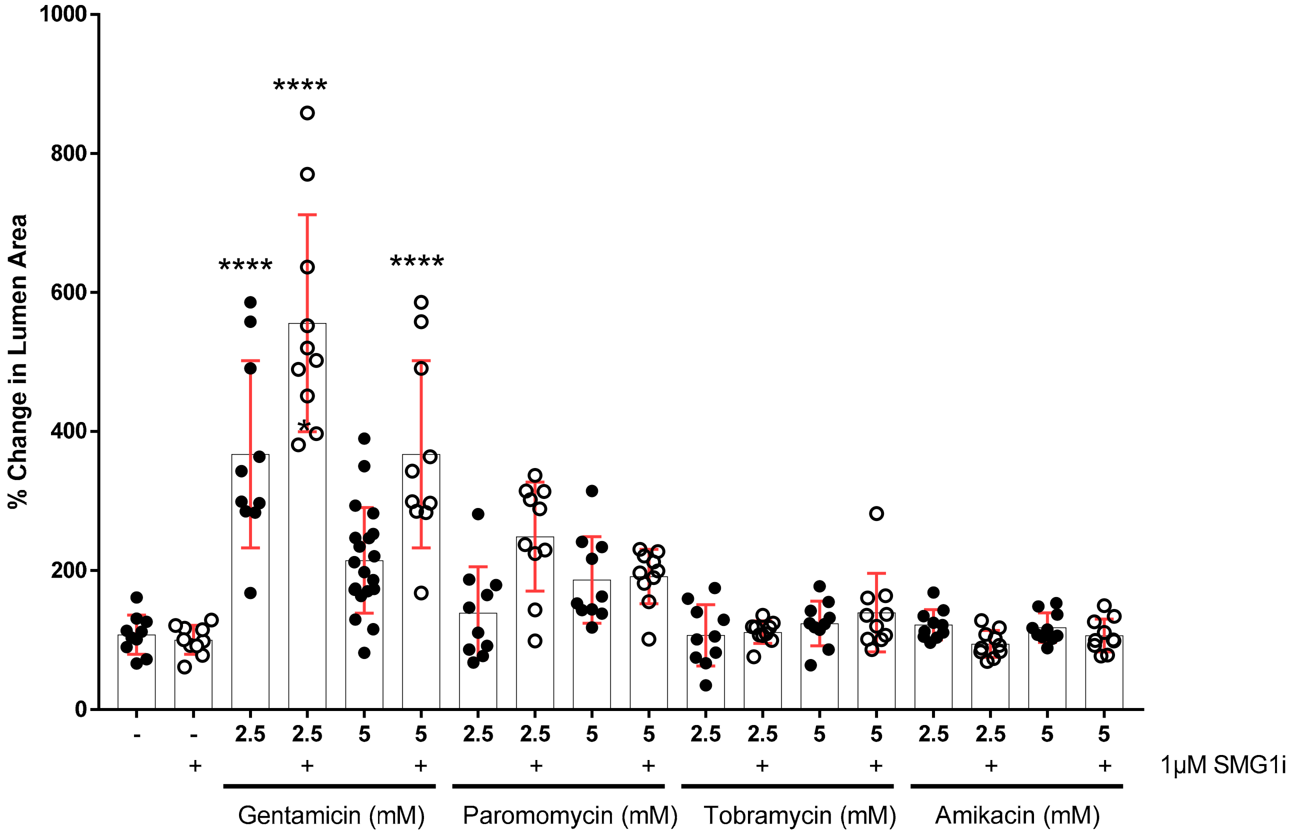


Supplementary Figure 5. Lumen measurements of alternative aminoglycosides and SMG1i. Percent change in lumen area at 180 minutes for indicated doses of aminoglycoside. Groups treated with 1μM SMG1i are indicated with the + sign. $n$=10 to 20 per treatment group. $p$****<0.0001.

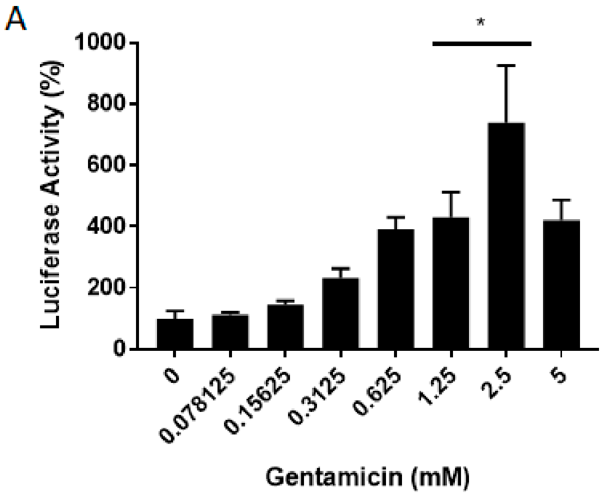
A
*
1000
800
600
400
200
0
Luciferase Activity (%)
0
0.078125
0.15625
0.3125
0.625
1.25
2.5
5
Gentamicin (mM)

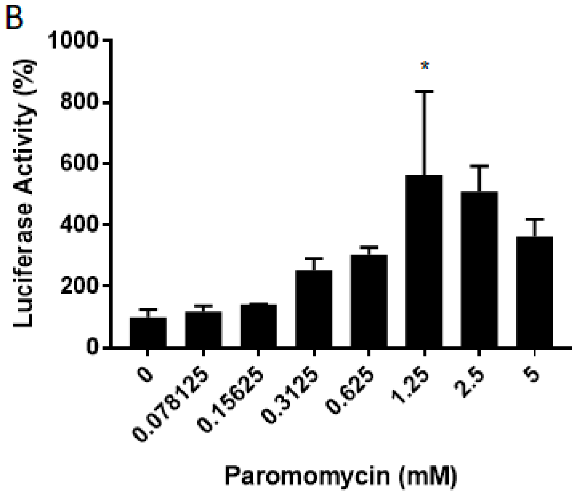
B
*
1000
800
600
400
200
0
Luciferase Activity (%)
0
0.078125
0.15625
0.3125
0.625
1.25
2.5
5
Paromomycin (mM)

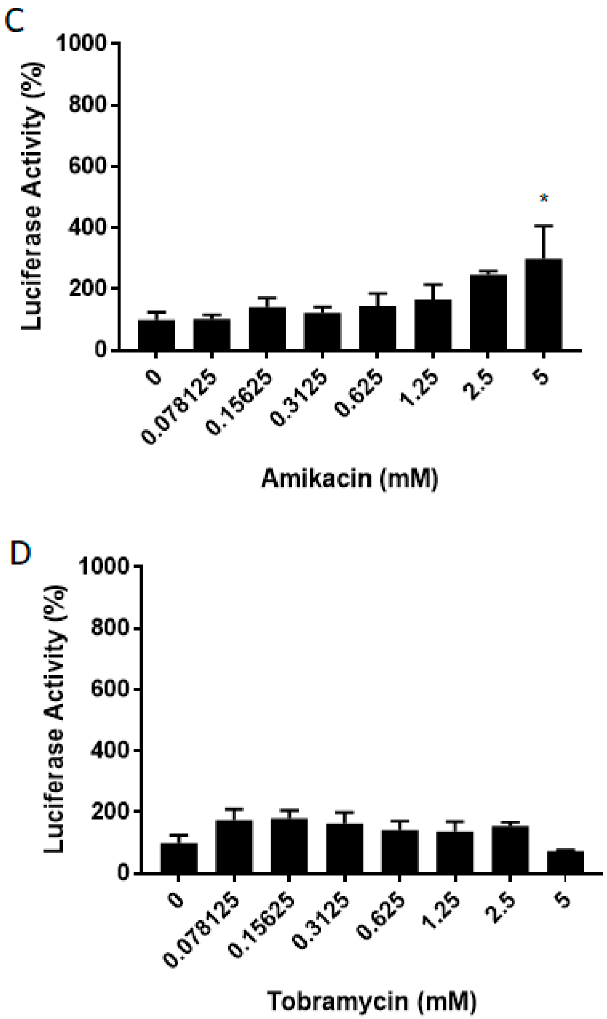


Supplementary Figure 6. Readthrough by non-G418 aminoglycosides measured by pFluc190UGA. Firefly luciferase activity in 3T3 fibroblast cells transfected with pFluc190UGA following a 24 hour incubation with the indicated doses of gentamicin (A), paromomycin (B), amikacin (C), or tobramycin (D). *n*=3 wells per treatment, *p**<0.05 vs 0 by one way ANOVA with post-hoc Tukey test ± SD.

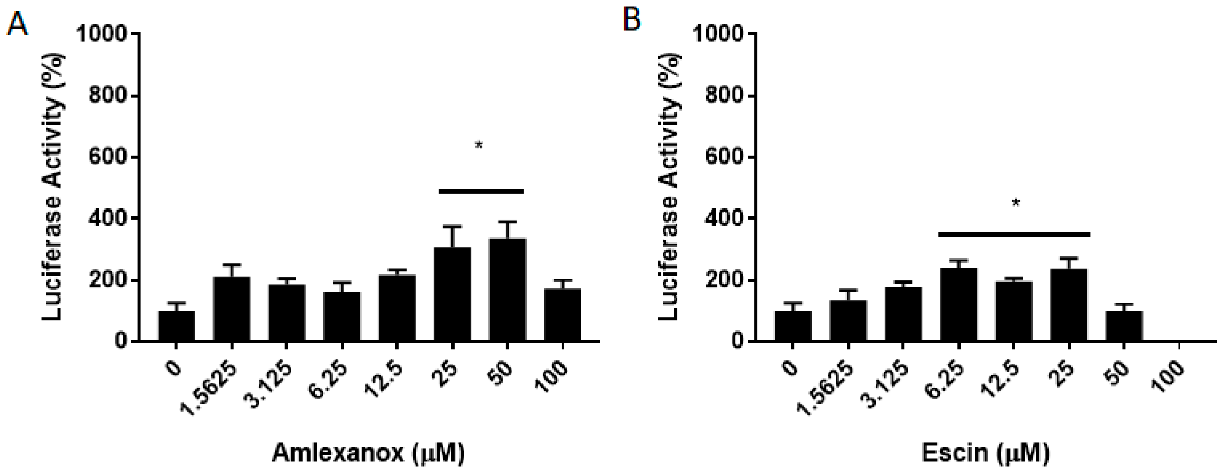


Supplementary Figure 7. Readthrough by non-aminoglycoside readthrough agents measured by pFluc190UGA. Firefly luciferase activity in 3T3 fibroblast cells transfected with pFluc190UGA following a 24 hour incubation with the indicated doses of amlexanox (A) or escin (B). *n*=3 wells per treatment group, *p**<0.05 vs 0 by one way ANOVA with post-hoc Tukey test, ± SD.

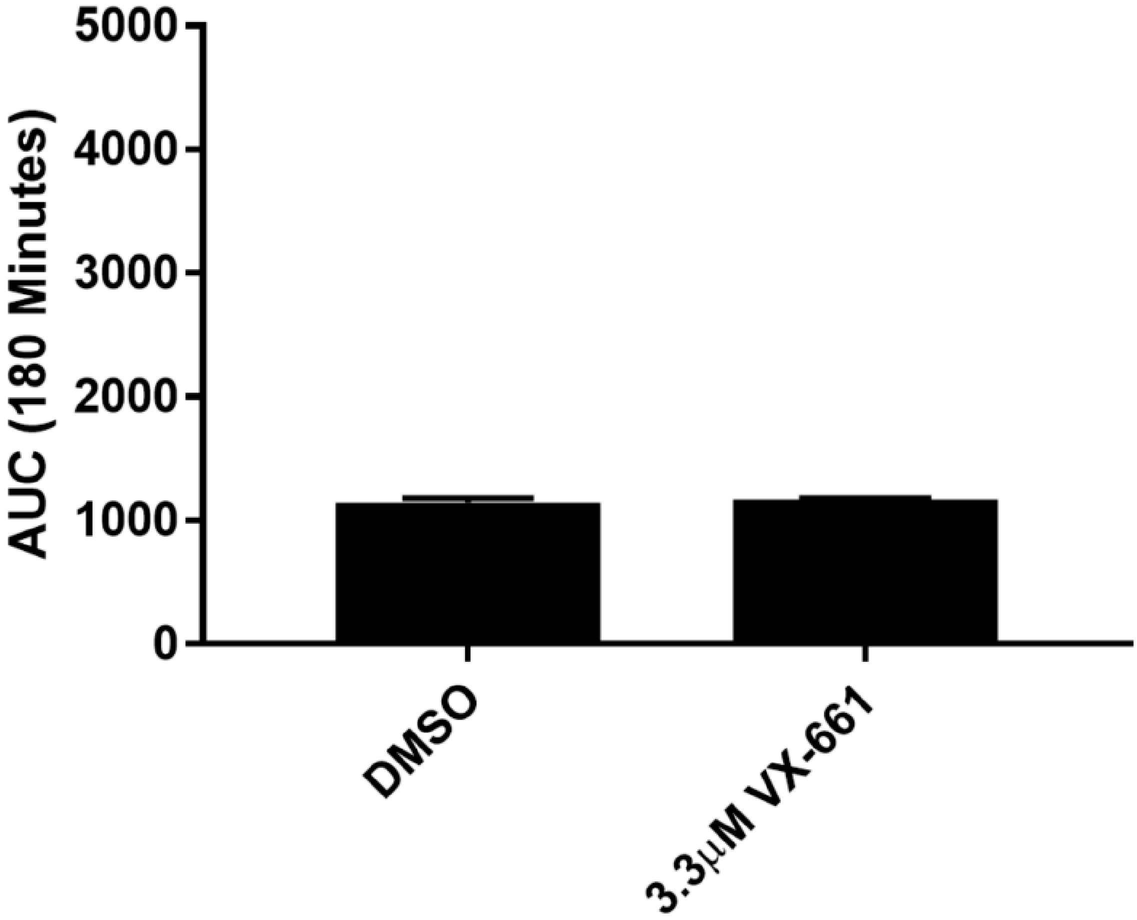


Supplementary Figure 8. VX-661 does not facilitate FIS independent of CFTR. AUC measurements from G542X intestinal organoids treated for 24 hours with DMSO or 3.3 μM VX-661. *n*=3.

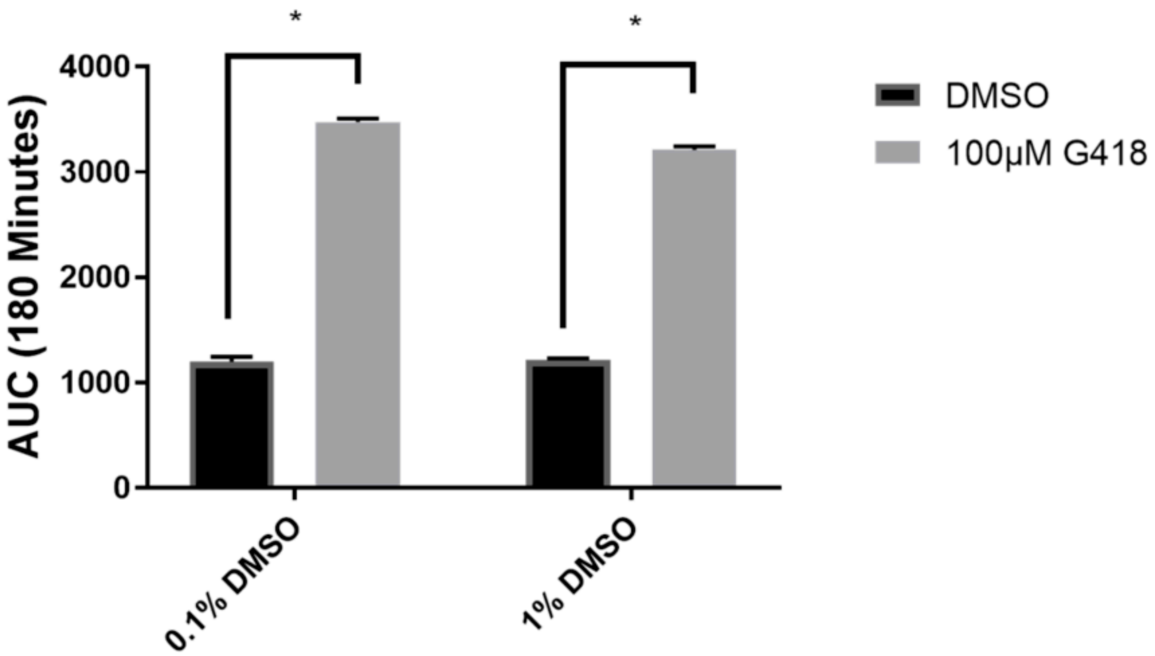


Supplementary Figure 9. 1% DMSO does not impede intestinal organoid FIS. AUC measurements from G542X intestinal organoids were incubated with the indicated compounds and either 0.1% or 1% DMSO. *p**<0.05 between indicated groups by two-way ANOVA with post-hoc Tukey test; *n*=3.
